# Facile Synthesis ZnS/ZnO/Ni(OH)_2_ Composites Grown on Ni Foam: A Bifunctional Materials for Photocatalysts and Supercapacitors

**DOI:** 10.1038/s41598-017-03200-2

**Published:** 2017-06-08

**Authors:** Jin Hao, Xiaobing Wang, Fanggang Liu, Shuang Han, Jianshe Lian, Qing Jiang

**Affiliations:** Key Laboratory of Automobile Materials, Ministry of Education, and Department of Materials Science and Engineering, Jilin University, Changchun, 130022 P.R. China

## Abstract

A facile one-step hydrothermal reaction was employed to synthesis an integrated bifunctional composite composed by a network structure of ZnS/ZnO/Ni(OH)_2_ nanosheets with ZnS/ZnO nanospheres *in situ* growing on Ni foam. The synergistic effect of these three substances make the composite having both improved electrochemical performances and photocatalytic activity. The ZnS/ZnO/Ni(OH)_2_-4mmol shows a high specific capacitance of 1173.8 F g^−1^ at 1 A g^−1^, as well as good rate capability and relatively stable cyclability. Using as photocatalyst, the methyl orange dye in solution can be completely decomposed under ultraviolet-visible radiation in about 80 min. And the composite is easy to be repeatedly used because bulk Ni foam was used as a carrier. Such a bifunctional composite material provides a new insight for energy storage and utilization as well as the water pollution treatment.

## Introduction

In this era of rapid development of science and technology, the demand for power sources is gradually increasing. Particularly, it is not always easy to meet the needs of both store a large amount of electric charges in small volumes and the ability of transferring charge quickly by a single energy storage device for modern mobile devices^1^. The study of traditional capacitor and battery has greatly promoted the development of electrochemical energy storage, which can be utilized for high power density and high energy density supply. As a potential design that combine these two main devices, supercapacitors, owning their fast charge and discharge speed and long cycle life^2^, has attracted extensive attention in recent years. However, it is inevitable to cause environmental pollution and destruction in the process of storage and use of energy, especially water pollutants caused by heavy metals and organic pollutants. On the other hand, agriculture, textile industry will generate residual organic pollutants and dyeing agents which inevitably leak into the water in the production process. How to effectively control the pollutants, especially the second pollution caused by the process of governance, is also an imminent task.

In recent years, a new idea of preparing bifunctional materials has gradually gained attention due to the critical demands in specific environments. Zhou *et al*. reported the use of functionalized metal-organic frameworks by inserting platinum ions in photosensitizers and hydrogen-evolution catalysts. This new metal-organic framework material could directly convert solar energy to chemical energy, which could be applied to solar energy conversion^3^. Yu *et al*. synthesized dual-doped molybdenum trioxide nanowires, which exhibited enhanced electrochemical properties for both fiber-shaped asymmetric supercapacitors and microbial fuel cells. This bifunctional anode material could be used for energy conversion and storage devices^4^. Thus it is possible to design new bifunctional materials with the function of both storing energy and treating sewage. Yet there are still limit reports about these kinds of bifunctional materials, due to the reliable semiconductor materials with good performance both in photocatalyst and supercapacitor have been rarely discovered.

So, our anticipation comes to the combination of two or more semiconductors. ZnO is an important wide-band gap semiconductor (3.37 eV), which has been applied as an ideal photocatalyst attributed to its relatively low price, environment friendly and carrier fluidity well^5, 6^. Meanwhile, ZnO as a template in preparing electrode materials could be utilized in the supercapacitor^7^. Analogously, owing to unique optical and electrical characteristics, ZnS always investigated as a photocatalyst and supercapacitor material^8, 9^. In addition, as a transitional element, Ni element has a variety of valance state, which makes Ni-based materials (especially for Ni(OH)_2_) possess high theoretical specific capacitance as well as well-defined redox behavior^10^. In order to enhance photocatalytic performance, Ni element is often used as doping elements to adjust the band gap structure of photocatalytic materials^11, 12^. Based on the above considerations, a bifunctional materials by combining ZnO, ZnS and Ni(OH)_2_ together was intend to be synthesized, which would be expected to bring good properties for both supercapacitor and photocatalyst.

It is well accepted that, the physical and chemical properties of the material are closely related to its structure. To date there are some common structures have been prepared including Zero-dimensional (0D) nanoparticles^13, 14^, one-dimensional (1D) nanoroads or nanowires^15, 16^, two-dimensional (2D) nanosheets^17, 18^, and three-dimensional (3D) nanostructures. Among them, 3D structured nano-materials have been widely explored ascribe to the large specific surface area and special structure^19, 20^. Especially the network structure of nanosheets has abundant porous with optimized pore size distribution, which would greatly improve the electrode/electrolyte contact area, provide more ion contact sites, reduce the volume, and shorten the ion diffusion process^21^. There have been a lot of reports on the design and research of 3D network structure. Zhu *et al*. successfully prepared 3D woven nanocomposite consisting of CoO nanoflowers and CNTs via a high-throughput hydrothermal reaction, herein flower-like nanostructure provided large surface area for electrochemical reaction^22^. Xie *et al*. reported the use of a 3D graphene network as a reactive template for porous MnO supercapacitor, This porous framework possessed an interconnected iron diffusion channel, which definitely raised its specific capacitance^23^. Zheng *et al*. synthesized a network-structured SnO_2_/ZnO heterojunction nanocatalyst through a simple two-step solvothermal method, the enhanced photocatalytic activity benefits from the pore structure with higher BET surface area, which could provide more surface reaction sites^24^. It is reported that powder photocatalysts are hard to be gathered and then affect its recycle in the process of governance of water pollution. So, designing active materials *in situ* grown on macroscopic Ni foam would overcome this problem effectively^25^. Moreover, Nickel foam substrate can be used not only as a reinforcement to enhance conductive performance and promote penetration of electrolytes in the practical application, but also as Ni source during the materials producing process^26–28^.

In this work, a Ni foam-ZnS/ZnO/Ni(OH)_2_ (for abbreviation it is called NZZN in the following) bifunctional composites were prepared on nickel foam substrates by one-step hydrothermal reaction. These three substances (ZnS, ZnO and Ni(OH)_2_) were combined together successfully and formed a porous nanosheet networks structure on the Ni foam surface. The morphology and composition of NZZN composites were characterized, and their electrochemical properties as well as photocatalytic activity were measured and evaluated.

## Results

**Figure 1:**
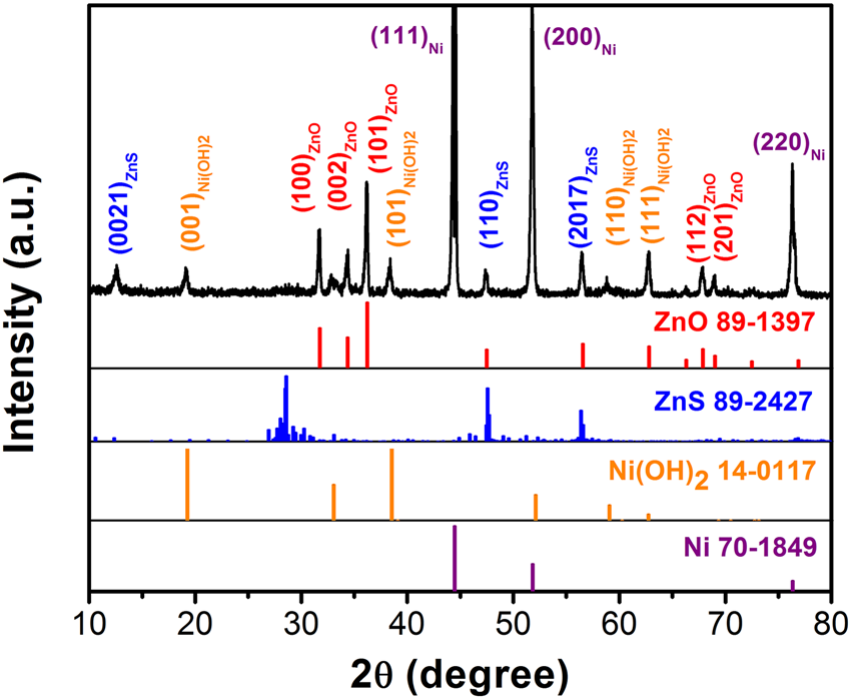
XRD patterns of the NZZN-4 mmol sample.

The crystalline structure and existing phase of the obtained NZZN-4mmol sample was characterized by X-ray diffraction (XRD) measurements, as shown in Fig. 1. The diffraction peaks of the two kinds of Zinc compound could be assigned to the (0021), (110), (2017) planes of ZnS (JCPDS card no. 89-2427) and the (100), (002),(101), (112), (201) plans of ZnO (JCPDS card no. 89-1397). The diffraction peaks correspond to the (001), (101), (110) and (111) planes of Ni(OH)_2_ (JCPDS card no. 14-0117) could also be indexed. In addition, the high intensity diffraction peaks correspond to the (111), (200) and (220) crystal planes could be well indexed to the Ni foam substrate (JCPDS card no. 70-1849). Thus, according to the XRD results, after the hydrothermal treatment, the composite structure consisting of three phases of ZnS, ZnO and Ni(OH)_2_ were successfully grown on the Ni foam substrate and no any other impurity phases was found.

**Figure 2:**
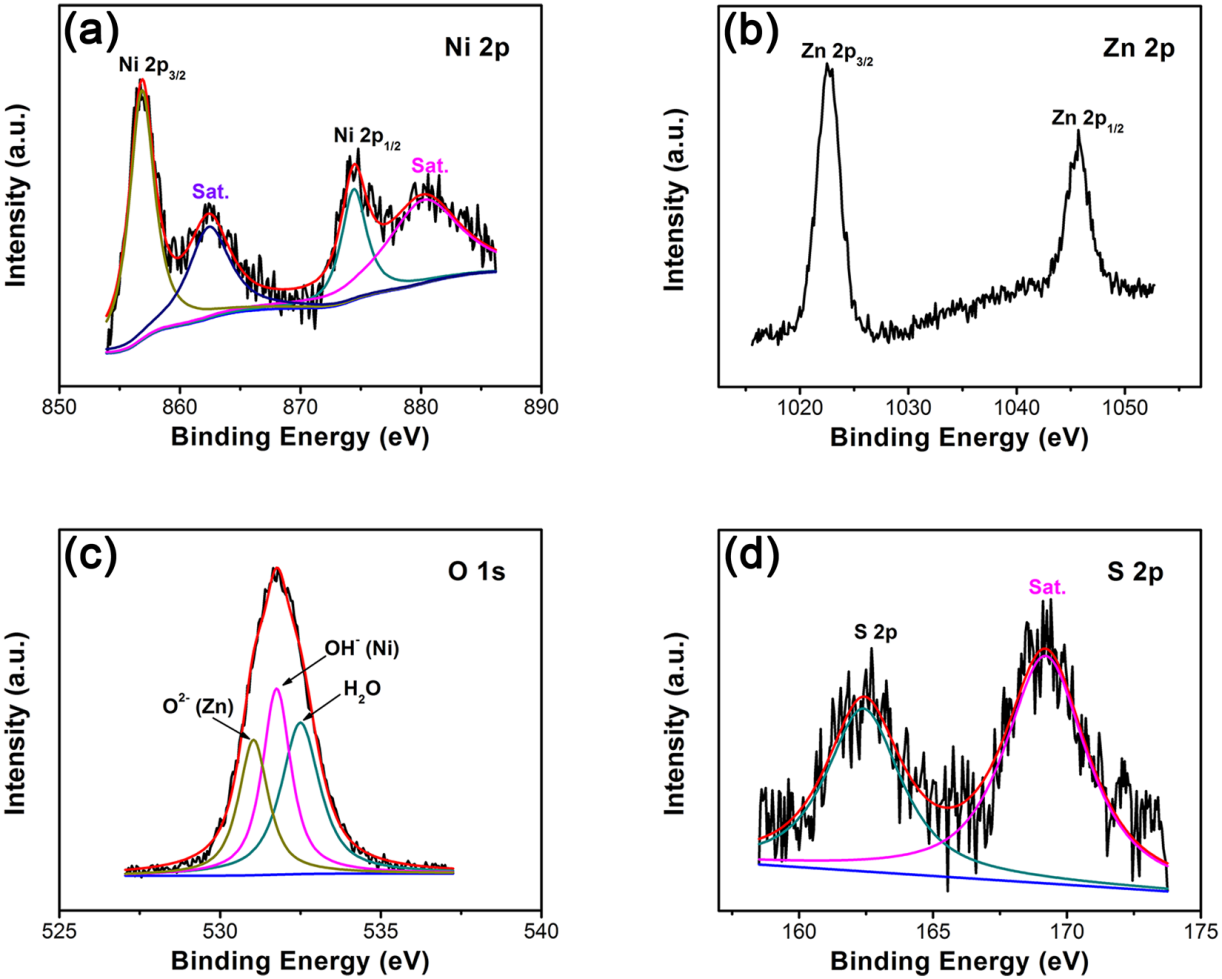
XPS spectra of the NZZN-4 mmol sample in the energy regions of (**a**) Ni 2p, (**b**) Zn 2p, (**c**) O 1 s and (**d**) S 2p.

X-ray photoelectron spectroscopy (XPS) measurements were carried out to investigate the surface elemental composition of NZZN-4 mmol sample, and the results were fitted by a Gaussian fitting method and shown in Fig. 2. For the Ni 2p spectrum in Fig. 2a, two main peaks with binding energy at 856.8 eV and 874.4 eV and the corresponding satellite (Sat.) peaks at 862.4 eV and 880.2 eV were found, respectively^29, 30^. In consideration of the above XRD result, it should be the Ni^2+^ in Ni(OH)_2_. Figure 2b shows the Zn 2p spectrum. The observed binding energy peaks at 1022.7 eV and 1045.5 eV are assigned to the Zn 2p_3/2_ and Zn 2p_1/2_ peaks of the Zn^2+^ ^31^. For the O 1 s spectrum in Fig. 2c, the binding energy at 531.0 eV and 531.76 eV are attributed to O^2−^(Zn) and OH^−^(Ni). Meanwhile, the binding energy at 532.5 eV is characteristic of H_2_O, which comes from crystal water and absorbed water from the air in samples. For the S 2p spectrum in Fig. 2d, two peaks at 162.4 eV and 169.2 eV could be fitted by a main peak and a Sat. peak of S^2−^ ^30^. So, the XPS results also confirm the formation of Ni(OH)_2_, ZnS, and ZnO on the Ni foam surface of the NZZN-4 mmol sample.

**Figure 3:**
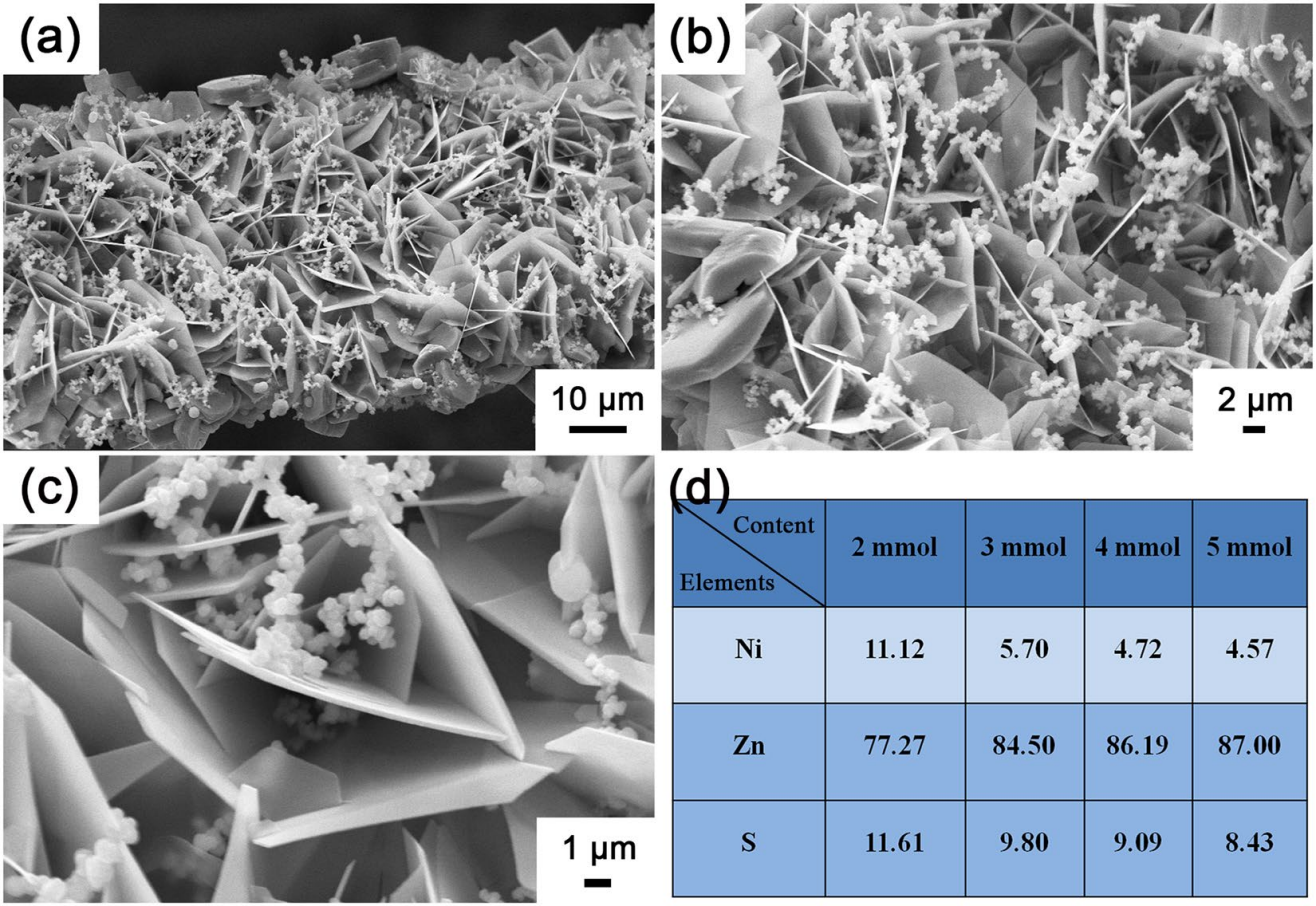
FESEM images of (**a**–**c**) NZZN-4 mmol. (**d**) The energy spectrum results contained Ni, Zn and S elements for NZZN-2 mmol, NZZN-3 mmol, NZZN-4 mmol and NZZN-5 mmol samples.

The morphologies and microstructures of ZZN were observed by utilizing FESEM, TEM and HRTEM. Figure 3a–c depicts the morphology of the NZZN-4 mmol through the FESEM images. As shown in Fig. 3a, it distinctly displays the ZZN consist of a large number of nanosheets and nanospheres *in situ* grown on the Ni foam framework successfully. Besides, these nanosheets interconnected with each other to form a network structure with lots of nanospheres dwelling in the space among the nanosheets. The higher magnification SEM images in Fig. 3b and c show the microstructure of the as-obtained sample in detail. It is clearly seen that the nanosheets were nearly perpendicular grew on the Ni foam substrate and form plenty of space among them with different angles. The nanospheres with uniform size about 500 nm–1 μm accumulate and distribute on the surface or in the interior of the nanosheet network. The energy spectrum was obtained from the surface of the nanosheets network, and the corresponding results can be seen in Fig. 3d. The major element is Zn, indicating that forming abundant of ZnO or ZnS. It is natural that the quantity of Zn increases with increasing Zn(NO_3_)_2_ concentration during the preparation, which as the Zn source. Furthermore, the contents of ZnS, ZnO and Ni(OH)_2_ in the composites was estimated as shown in Table S1, and the results show ZnO > ZnS > Ni(OH)_2_. Supplementary Fig. S1 shows FESEM images of other samples of NZZN-2 mmol, NZZN-3 mmol and NZZN-5 mmol, suggesting the density of composites increases with Zn content in the sample.

**Figure 4:**
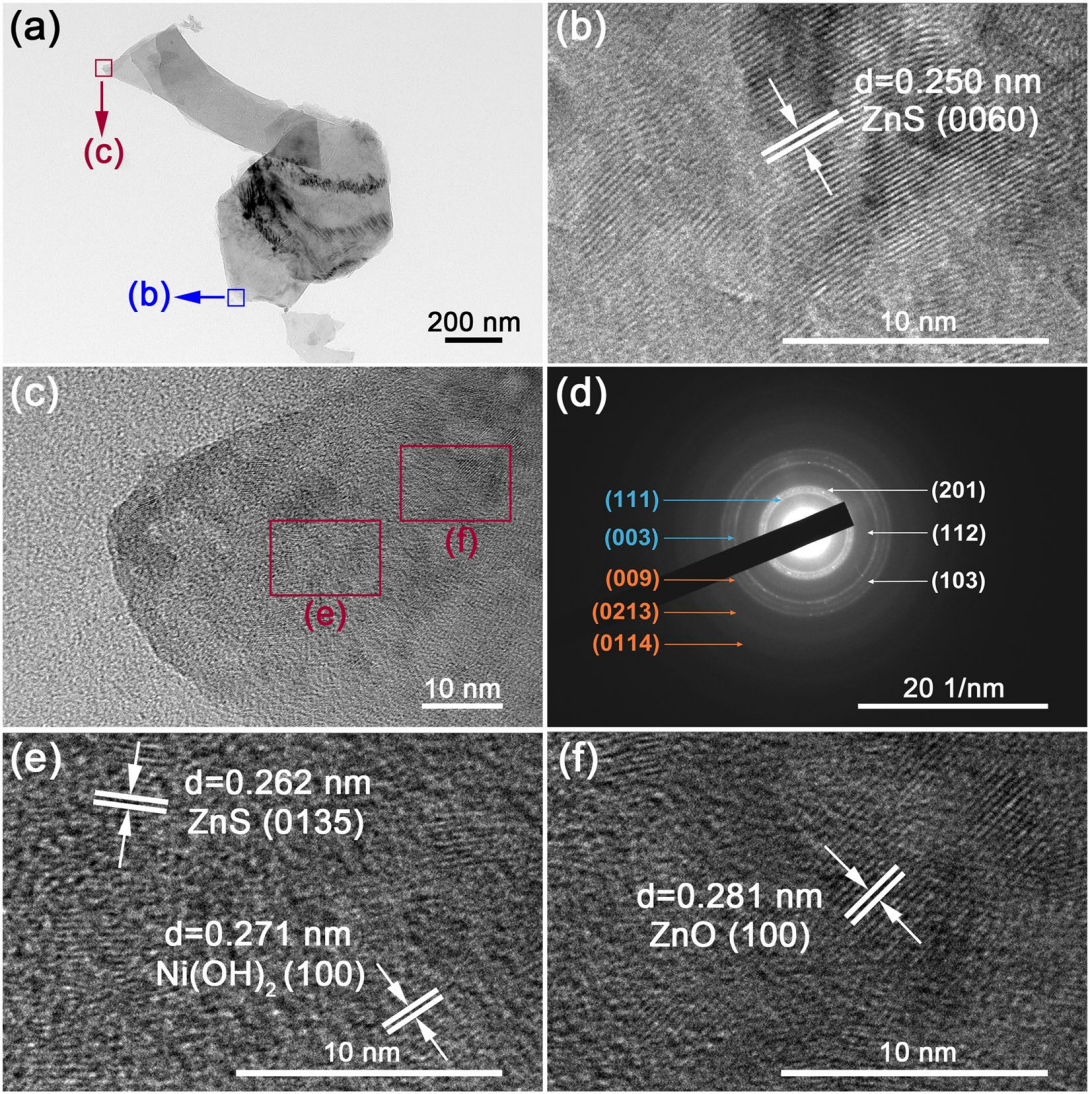
TEM characteristics of the nanosheets in NZZN-4 mmol. (**a**) Low magnification TEM image, (**b**,**c**,**e**,**f**) HRTEM images and (**d**) SAED patterns. The HRTEM images in (**b**,**c**) represent the corresponding crystalline characteristics of the blue and red square areas in (**a**), respectively; the HRTEM images in (**e**,**f**) show the close-up views in (**c**); in the (**d**), white tabs index the ZnO, blue tabs index the Ni(OH)_2_ and orange tabs index the ZnS.

Figure 4a depicts the low magnification TEM image of NZZN-4 mmol ultrasonically exfoliated nanosheets. The relatively darker part in the lower right position is the root segment of NZZN-4 mmol located between nanosheets and Ni foam, and an individual nanosheet grown on it. The HRTEM images recorded from the blue and red square areas in Fig. 4a are shown in Fig. 4b and c, respectively. As is shown, Fig. 4b is recorded from the blue square areas in Fig. 4a, shows a fringe spacing of 0.250 nm, indexed to the (0060) plane of ZnS, demonstrating that the main component of the root segment is ZnS. Figure 4c is recorded from the red square areas in Fig. 4a, and the crystal characteristics of two red square areas in Fig. 4c are demonstrated by Fig. 4e and f. In Fig. 4e, the interplanar spacings of 0.262 and 0.271 nm can be assigned to (0135) plane of ZnS and (100) plane of Ni(OH)_2_. In Fig. 4f, another local zone on the nanosheet, the lattice fringe of 0.281 nm is in good agreement with the (100) plane of ZnO. Furthermore, the selected area electron diffraction (SAED) pattern taken on the nanosheets is shown in Fig. 4d, the indexed diffraction rings of (201), (112) and (103) correspond to ZnO, (111) and (003) correspond to Ni(OH)_2_, as well as (009), (0213) and (0114) correspond to ZnS. So, the nanosheet is consisted of ZnS, ZnO and Ni(OH)_2_. The continuous rings without light spots on the SAED pattern also indicates that all these three ingredients are very small nanocrytals bounded together.

**Figure 5:**
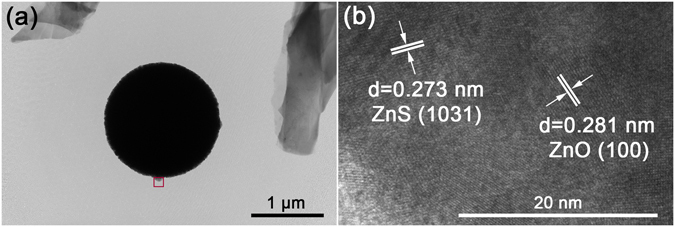
(**a**) TEM image and (**b**) HRTEM image of the nanospheres in NZZN-4 mmol. The crystalline details in red square area in (**a**) are described in (**b**).

The TEM image of the nanospheres in NZZN-4 mmol is shown in Fig. 5a, the details in red square area is illustrated by HRTEM, shown in Fig. 5b. The lattice fringes of 0.273 nm and 0.281 nm in the HRTEM image can be assigned to the (1031) plane of ZnS and (100) plane of ZnO, respectively. So, the nanospheres are the composite of ZnS and ZnO.

Based on the above experimental results, a possible formation mechanism of the NZZN-4 mmol 3D nanostructure can be stretched out. During the hydrothermal process, thioacetamide (TAA) was used as Sulfur source in solution slowly decomposed to H_2_S, CH_3_COO^−^ and NH_4_^+^^32^. The H_2_S reacted with the exposed Ni foam to form Ni^2+^ cations. The released Ni^2+^ in the solution could form Ni(OH)_2_ through reacting with OH^−^ which comes from CH3COO^−^ hydrolyzation^27, 33^. The decomposed H_2_S reacted with Zn(NO_3_)_2_ in the solution to form ZnS. Some Zn^2+^ would precipitate out to form Zn(OH)_2_ and then continue decomposed into ZnO by heating^34^. The quantities of ZnS and ZnO were higher than that of Ni(OH)_2_, because there were more Zn^2+^ than Ni^2+^ around the surface of Ni foam. The reaction process is summarized as follows:

1 $$C{H}_{3}CSN{H}_{2}+2{H}_{2}O\to {H}_{2}S+C{H}_{3}CO{O}^{-}+N{H}_{4}^{+}$$

2 $$C{H}_{3}CO{O}^{-}+{H}_{2}O\to C{H}_{3}COOH+O{H}^{-}$$

3 $$Ni+{H}_{2}S+{H}_{2}O\leftrightarrow NiH{S}_{ads}+{H}_{3}{O}^{+}+{e}^{-}$$

4 $$NiH{S}^{+}+{H}_{3}{O}^{+}\to N{i}^{2+}+{H}_{2}S+{H}_{2}O$$

5 $$N{i}^{2+}+2O{H}^{-}\to Ni{(OH)}_{2}$$

6 $$Z{n}^{2+}+{H}_{2}S\to ZnS+{H}_{2}$$

7 $$Z{n}^{2+}+2O{H}^{-}\to Zn{(OH)}_{2}$$

8 $$Zn{(OH)}_{2}\to ZnO+{H}_{2}O$$

## Discussion

**Figure 6:**
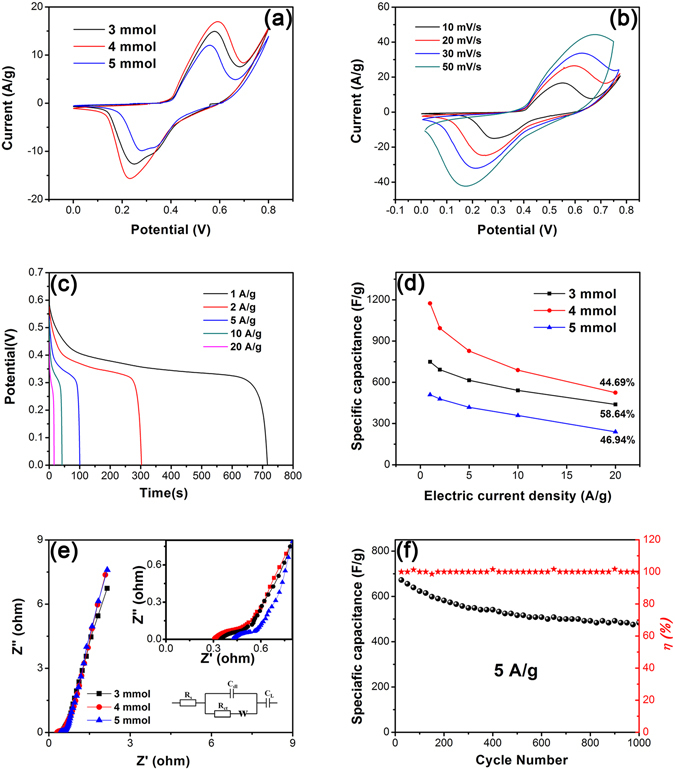
(**a**) Comparison of CV curves of NZZN-3 mmol, NZZN-4 mmol and NZZN-5 mmol electrodes at a scan rate of 10 mV s^−1^; rate performance curves (**b**,**c**) of NZZN-4 mmol electrode; (**d**) rate capacitance calculated by galvanostatic charge-discharge curves, and (**e**) EIS with inset showing the close-up views of high-frequency region of NZZN-3 mmol, NZZN-4 mmol and NZZN-5 mmol electrodes, and the electrical equivalent circuit used for fitting impedance spectra. (**f**) Cycling performances and coulombic efficiency of NZZN-4 mmol electrode at a current density of 5 A g^−1^ for 1000 cycles.

The supercapacitor performances were studied with NZZN-3 mmol, NZZN-4 mmol and NZZN-5 mmol samples as work electrode in a three-electrode system by means of cyclic voltammetry (CV) and galvanostat charge-discharge tests. The CV curves at the same scan rate of 10 mV s^−1^ are shown in Fig. 6a. The CV curve of NZZN-4 mmol electrode also covers the largest area demonstrating it possesses highest specific capacitance among the three tested samples. Then, the NZZN-4 mmol sample were measured at a series of scan rates from 10 mV s^−1^ to 50 mV s^−1^ and the results are shown in Fig. 6b. As we can see, all CV curves within the potential windows ranging from 0 to 0.8 V display a similar shape and reveal a regularity that the anodic peaks shift towards positive potential and the cathodic peaks shift towards negative potential respectively with the scan rates gradually upgrade, which is ascribed to the strengthened electric polarization happening when the scan rate increased^35^. Figure 6c shows the galvanostatic charge-discharge curves of NZZN-4 mmol at different current densities from 1 A g^−1^ to 20 A g^−1^. Pronounced potential platforms are observed from charge-discharge curves which are consistent with the above CV test results, suggesting that the NZZN-4 mmol electrode exhibits a decent pseudocapacitance behavior. Similar rate performance tests of NZZN-3 mmol, NZZN-5 mmol electrodes were also done and the results are observed in Supplementary Fig. S2, which kept the same regularities as that of NZZN-4 mmol. As shown in Fig. 6d, based on the galvanostatic charge-discharge curves executed at various current densities, it is seen that the calculated rate capacitance values of NZZN-4 mmol electrode are 1173.8, 993.4, 827.9, 688.5 and 524.6 F g^−1^ at 1, 2, 5, 10, and 20 A g^−1^, respectively. Compared with other samples, NZZN-4 mmol still possesses larger specific capacitance value among these the samples at the same current densities, indicating that NZZN-4 mmol sample has the excellent supercapacitor performance.

In order to get more information about the basic behavior between the electrolyte and electrode, the EIS measurements were conducted at a frequency range of 10^−2^ to 10^5^ Hz and its data were analyzed by ZSimpWin software, as shown in Fig. 6e. In general, all impedance plots consist of two partially overlapped semicircles in the high-frequency region and medium-frequency regions and an inclined line in the low-frequency region^36^. An equivalent circuit used to fit the impedance curve is given in the Fig. 6e, which is similar to the circuit employed for the working electrode of supercapacitors. The EIS spectrum can be fitted from the redox process of NZZN electrode and the calculated results by ZSimpWin software are shown in Table S2. At high frequency (the inset in Fig. 6e), it can be observed that the intercept on the Z real axis, represents bulk solution resistance R_s_ are very small for NZZN-3 mmol, NZZN-4 mmol and NZZN-5 mmol electrodes. In addition, the semicircle region in the impedance curve corresponds to R_ct_ (also called Faraday resistance) at the electrode/electrolyte interface^37^. The calculated results shows that NZZN-4 mmol has the lowest R_ct_ value 0.0887 Ω, while R_ct_ value of NZZN-3 mmol is 0.0909 Ω and that of NZZN-5 mmol is 0.0938 Ω.

Herein, cycle performance of NZZN-4 mmol electrode was measured by consecutive galvanostat charge-discharge cyclic tests at room temperature at a current density of 5 A g^−1^, and the cycling life relatively data are shown in Fig. 6f. A decreasing tendency of the specific capacitance is observed, which might ascribe to the weak adhesion between the active material and the substrate. After 1000 cycles, it can still maintain 72% specific capacitance retention and the coulombic efficiency stay at about 100% all the time, which evidently showing a good cycling performance and utilization potentiality.

**Figure 7:**
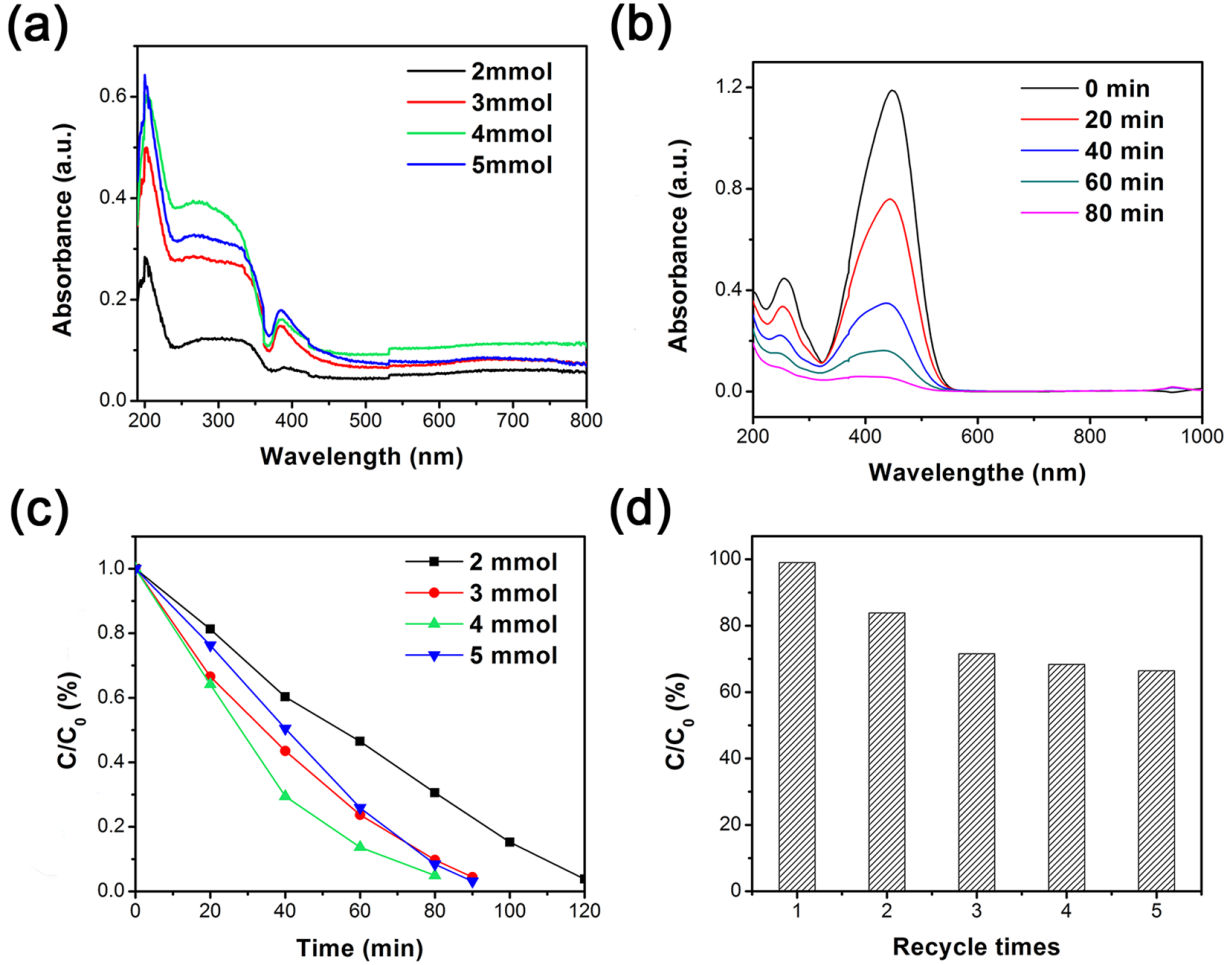
(**a**) UV-visible spectra of NZZN-2 mmol, NZZN-3 mmol, NZZN-4 mmol and NZZN-5 mmol samples; and (**b**) photocatalytic degradation efficiency of MO aqueous solution containing different samples: NZZN-2 mmol, NZZN-3 mmol, NZZN-4 mmol and NZZN-5 mmol as a photosensitizer. (**c**) UV-vis absorption spectra of MO solution with as-obtained NZZN-4 mmol sample. (**d**) Five cycles for photodegradation of MO for NZZN-4 mmol sample.

The UV-vis absorption spectra of the NZZN-2 mmol, NZZN-3 mmol, NZZN-4 mmol and NZZN-5 mmol are shown in Fig. 7a. Compared with other samples, the NZZN-4 mmol displays a stronger absorption in the UV and visible light regions, which suggests the NZZN-4 mmol exhibits better capacity for optical absorption. Based on such results, the photocatalytic performances were evaluated by methyl orange (MO) decomposition tests.

Figure 7b depicts the time-dependent degradation process of UV-vis absorption spectrum variation upon photodecomposition of MO over the NZZN-4 mmol sample. As shown in Fig. 7b, the absorption peak at 464 nm gradually weakened and the curve shifts down with time passed, and the maximum absorbance decreases nearly to zero after irradiation for 80 minutes, meaning the MO can be effectively degraded by NZZN-4 mmol within 80 minutes. The experimental tests of NZZN-2 mmol, NZZN-3 mmol, NZZN-5 mmol samples under the same conditions were also taken, and the corresponding results can be found as Supplementary Fig. S3. For comparison, the photocatalytic degradation process for four samples are summarized and shown in Fig. 7c. It is seen that MO can be effectively degraded after UV-vis light illumination by all these samples and NZZN-4 mmol presents the highest photocatalytic activity, with 95.1% MO being degraded in 80 minutes. The time-dependent degradation curves of NZZN-4 mmol, Ni(OH)_2_ and ZnS/ZnO catalyst under UV-vis light irradiation are shown in Fig. S4, and NZZN-4 mmol shows much higher photocatalytic efficiency than the other two photocatalysts. The reusability of the NZZN-4 mmol sample was evaluated by a five-cycle test which was performed for degradation of MO under UV-vis irradiation for 80 minutes; the results are shown in Fig. 7d. The high photocatalytic activity of the NZZN-4 mmol sample gradually decreased in the first three cycles (from 95.1% to about 70%) and then keep relatively stable for the last two tests. The decrease of photocatalytic activity in these cycles might due to one of sample’s ingredient ZnS lost some of its active sites during the photocatalytic reactions^38^. Therefore, the cycle reusability is an evident advantage of the present composite over the photocatalytes in form of nano or micro powder which is difficult to be recycling used.

The composite NZZN-4 mmol has the best capacitance value and the best photocatalytic activity. This can be ascribed to its microstructure and composition. The FESEM results showed that the nanosheet networks with nanospheres are getting more and more intensive as the content of Zn(NO_3_)_2_ increases. At 4 mmol, it arrives to a optimum combination among porosity, density and even distribution of the nanosheet networks and nanospheres. The content of Zn also reaches to its nearly maximum (see Fig. 3d), as ZnO and ZnS are the main ingredients contributing to the electrochemical performance in both capacitance ability and photocatalytic activity. Further increasing the content of Zn(NO_3_)_2_ in preparing process will not continuously increase the content of Zn, but intend to increase the paricle size and decrease the even distribution of them, so the performance is reduced. Thus there is a relatively optimum composition (4 mmol Zn(NO_3_)_2_) of the material to achieve the best performances as supercapacitor electrode and in photocatalytic degradation of contaminants.

**Figure 8:**
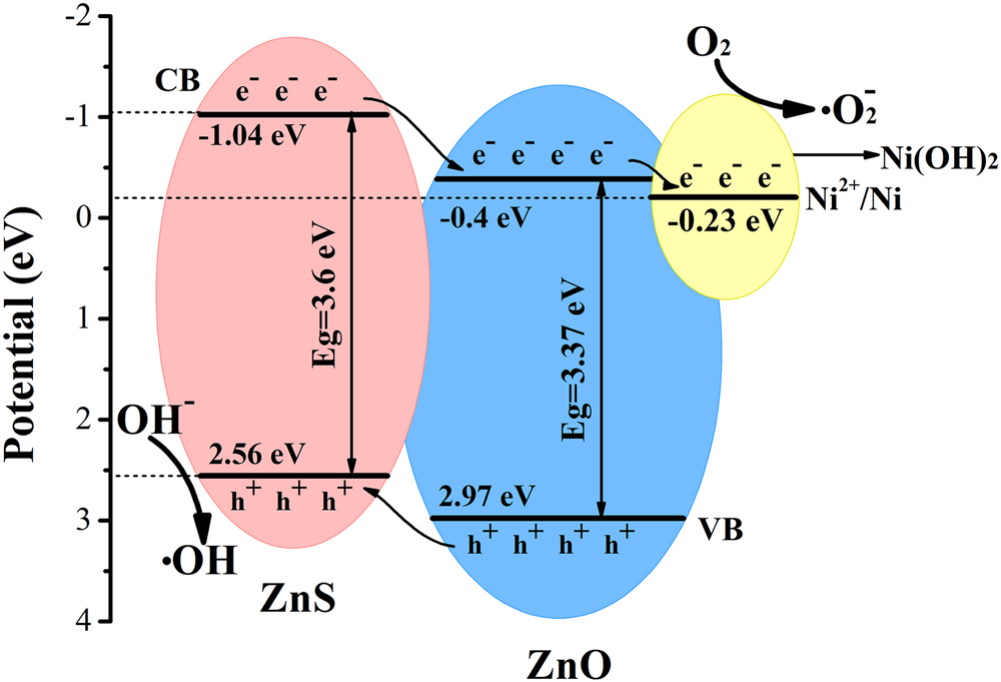
Schematic illustration for photodegradation mechanism, and the charge transfer and separation in the NZZN under UV-vis light irradiation.

In order to further explore the advantage of NZZN composites work as a photocatalyst, a possible photocatalytic mechanism was proposed according to the experimental results and previous literature reports, as shown in Fig. 8. For ZnS and ZnO, because the conduction band (CB) and valence band (VB) of ZnS lies on a more negative potential than that of ZnO, the photo-excited electrons in the CB of ZnS can transfer to that of ZnO, and the holes on the VB of ZnO can simultaneously transfer to that of ZnS under the UV-vis light irradiation^39^. It is worth noting that the potential (−0.23 eV) of Ni^2+^/Ni is further less negative than the CB level of ZnO, thus photostimulated electrons in the CB of ZnO can transfer to Ni(OH)_2_ and then effectually reduce some Ni^2+^ to Ni^0^ atoms, finally forming Ni atoms or clusters. The produced Ni atoms or clusters can act as a co-catalyst to promote the separation and transfer of photogenerated electrons from CB of ZnO to Ni(OH)_2_/Ni clusters^40^. As a result, the lifetime of photogenerated electrons and holes formed on the surface of NZZN could be prolonged. The photogenerated electrons will react with electronic acceptors, like absorbed O_2_ to form a superoxide anion radical (·O_2_^−^). Accordingly, the photogenerated holes moved from VB of ZnO will be trapped by OH^−^ to produce high hydroxyl radical species (·OH), which can participate in decomposing organic chemicals as a strong oxidant^41^.

Thus, the obtained electrochemical capacitive behaviors and enhanced photocatalytic activity of NZZN-4 mmol might be ascribed to the following reasons: (I) the nanosheets consist of three kinds of phases: ZnS, ZnO and Ni(OH)_2_, which have a good synergistic effect such as the fast electron transfer and electrolyte diffusion among them in the reactive process of either supercapacitor or photocatalysis; (II) the porous networks structure can accelerate the diffusion of electron and ion due to the interconnections between the nanosheets. In addition, the 3D networks structure composed by nanospheres and nanosheets could provide more active sites per unit volume, which is advantageous to promote the redox reactions; (III) the Ni foam is a conductive substrate which strongly enhanced the conductivity of the NZZN electrodes. In a word, ZnS and ZnO play major roles in the electrode reaction and photocatalysis, meanwhile, ZnS can provide capacitance in electrochemical performance. Ni(OH)_2_ is also the main provider of capacitance. In photocatalysis, ZnO and ZnS are used as a common catalyst due to their suitable band gap. Ni(OH)_2_ not only acts as a light absorption in the reaction process. The Ni foam is mainly used for the rapid conduction of electrons; it can be used as a current collector also can improve the conductivity of the composite in the supercapacitor. Ni foam favors the rapid separation of electron-hole pairs in the photocatalysis.

## Conclusion

In summary, we synthesized a ZnS/ZnO/Ni(OH)_2_ composite with 3D nanosheet networks structure *via* an one-step hydrothermal reaction, which is a promising bifunctional material in photocatalyst and supercapacitor. When the ZnS/ZnO/Ni(OH)_2_ composite (NZZN-4 mmol) applied as a photocatalyst, it displays enhanced photocatalytic activity in the degradation of MO under UV-vis light irradiation. Besides, when employed it as an electrode material in supercapacitor applications, the nanostructured composite exhibits a good electrochemistry properties. It shows the highest specific capacitance of 1173.8 F g^−1^ and 524.6 F g^−1^ at a lower density of 1 A g^−1^ and a higher density of 20 A g^−1^, respectively. Additionally, the capacitance can maintain 72% of its initial value after 1000 cycles at a current density of 5 A g^−1^. The method using Ni foam as porous substrate to *in-situ* synthesize 3D nanostructured composite containing three compounds (ZnS/ZnO/Ni(OH)_2_) may provides a new strategy to obtain bifunctional materials to cover even more various needs.

## Methods

### Chemicals and the synthesis of NZZN

All solvents and chemical regents in the present work were of analytic grade and used without further purification.

Ni foam was used as matrix sample. First, it was successively immersed and ultrasonic treated in the acetone, hydrochloric acid and ethanol for 20 minutes, respectively, to remove the surface impurities and oxide layers, then the Ni foam was rinsed with ethanol for three times and dried in vacuum at 60 °C.

**Figure 9:**
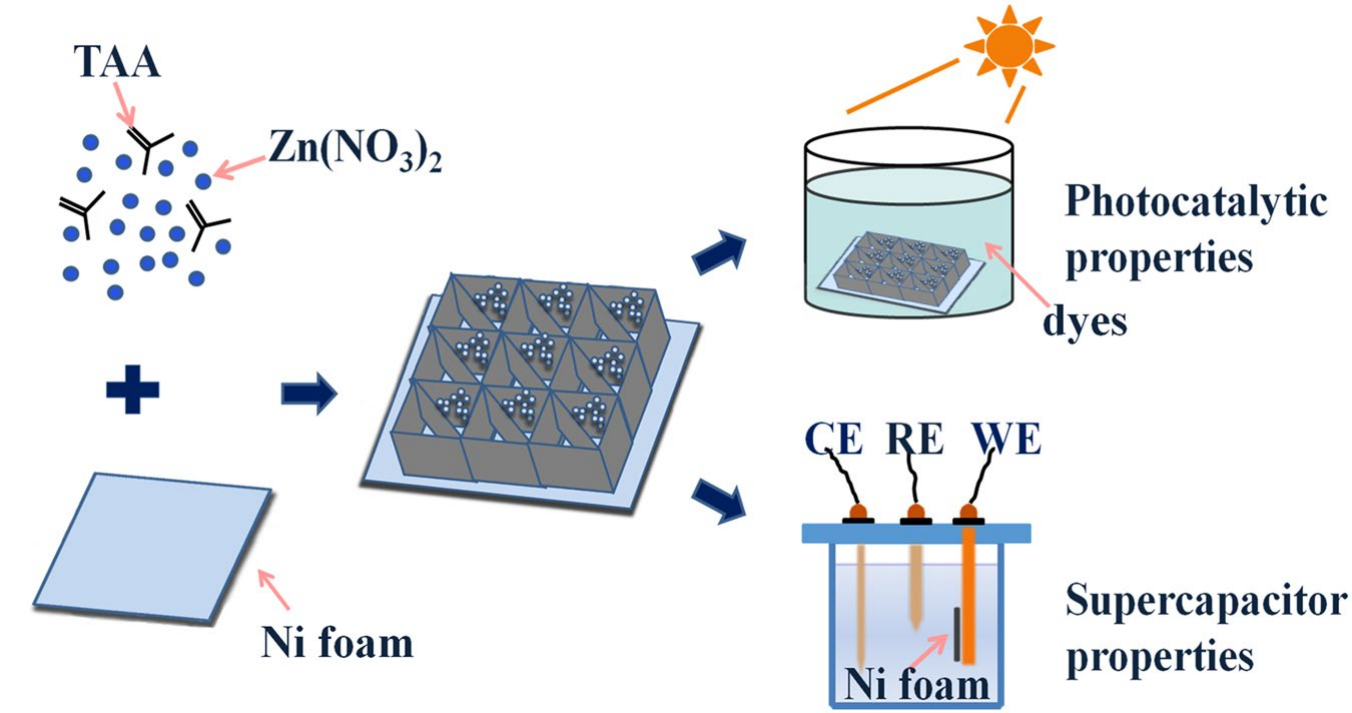
Schematic illustration of the formation mechanism of NZZN.

Subsequently, the pretreated Ni foam was cut into pieces of size 2 cm × 3 cm and each piece was weighed. Thioacetamide (TAA, 37.5 mg) and Zn(NO_3_)_2_ (4 mmol) were dissolved in the mixture solution of deionized water (15 mL) and ethanol (15 mL), with stirring for 15 minutes. The mixed solution was transferred into a 50 mL of Teflon-lined stainless steel autoclave and then put one piece of Ni foam into this autoclave. The sealed autoclave was then heated at 180 °C for 6 h for hydrothermal reaction. After the autoclave cooled to room temperature naturally, the as-synthesized sample was washed with deionized water several times and dried at 60 °C for 12 h, and then the prepared sample was weighed and labeled as NZZN-4 mmol. Samples with different quantity of Zn(NO_3_)_2_ (2 mmol, 3 mmol, and 5 mmol) were also prepared and they were marked as NZZN-2 mmol, NZZN-3 mmol and NZZN-5 mmol, respectively. The design, formation and possible function are schematically illustrated in Fig. 9.

In order to further compare the characteristics, Ni(OH)_2_/Ni foam and ZnS/ZnO/Ni foam composites were prepared through similar methods.

### Preparation of the Ni(OH)_2_

The pure Ni(OH)_2_/Ni foam was obtained through twice hydrothermal process. First, TAA (37.5 mg) dissolved in the mixed solution of deionized water (15 mL) and ethanol (15 mL) with stirring for 15 minutes, followed by the mixed solution with Ni foam (2 × 3 cm^2^) was sealed in a 50 mL of Teflon-lined stainless steel autoclave, kept at 180 °C for 6 h and then cooled to room temperature. The as-synthesized sample was washed with deionized water several times and dried in vacuum at 60 °C, and then denoted as Ni_3_S_2_/Ni(OH)_2_. The prepared sample was subjected to a hydrothermal reaction again at 180 °C for 6 h with 30 mL deionized water. The retreated sample was washed with deionized water several times and dried at 60 °C for 12 h. The obtained sample marked as Ni(OH)_2_-6h.

In order to obtain a more pure substance, the hydrothermal reaction temperature is 6 h, 12 h and 24 h, respectively, which were marked as Ni(OH)_2_-X (X = 6 h, 12 h and 24 h). The appropriate time was selected according to the XRD test results which were shown in Fig. S5.

### Synthesis of ZnS/ZnO

The ZnS/ZnO/Ni foam composites were obtained through a one-step hydrothermal process. TAA (37.5 mg) and Zn(NO_3_)_2_ (4 mmol) were dissolved in the mixture solution of deionized water (15 mL) and ethanol (15 mL) with stirring to obtain a mixed solution, and then was transferred into a 50 mL autoclave. The autoclave was sealed and heated at 180 °C for 6 h, and labeled as ZnS/ZnO. The precipitates were centrifuged at 8000 rpm with deionized water and ethanol repeated washing, then dried at 60 °C for 12 h and labeled as ZnS/ZnO.

### Characterizations

Powder X-ray diffraction (XRD) data of the as-obtained samples were recorded on a Rigaku D/max 2500PC diffractometer employing within 2θ in the range of 10–80° and Cu Kα radiation (λ = 1.54156 Å) at a scan rate of 4° min^−1^. X-ray photoelectron spectroscopy (XPS) spectra were obtained on an ESCALAB Mk II (Vacuum Generators) spectrometer with unmonochromatized Al Kα X-rays (240 W). Cycles of XPS measurements were conducted in a high-vacuum chamber with a base pressure of 1.33 × 10^−6^ Pa. Morphologies and structure observation of the as-prepared samples were determined using JSM-6700F field emission scanning electron microscopy (FESEM), JEM-2100F transmission electron microscopy (TEM), high-resolution TEM (HRTEM) and selected area electron diffraction (SAED), ultraviolet-visible (UV-vis) spectrophotometer. The electrochemical measurements were carried out in a PMC-1000 electrochemical workstation.

### Electrochemical measurements

For electrochemical studies, the as-obtained NZZN with the same area (1 cm^2^) was used as work electrode measured in a three-electrode system and with 3 M KOH aqueous solution. The calomel electrode and platinum sheet was used as reference electrode and counter electrode, respectively. The cyclic voltammograms (CVs), galvanostatic charge-discharge curves and electrochemical impedance spectroscopy (EIS) measurements were performed to investigate electrochemical properties by a PCM-1000 electrochemical workstation.

### Photocatalytic activity measurements

The photocatalytic performance of the NZZN was tested by degrading 20 mg/L methyl orange (MO). First, a piece of the as-obtained NZZN sample (2 cm × 1 cm) was immersed into 20 mL MO and the solution was placed in dark room for 30 minutes at room temperature to make sure an adsorption-desorption equilibrium being reached. Then the mixed solution (containing about 18 mg ZnS/ZnO/Ni(OH)_2_ catalyst and 20 mL MO) was placed at a distance of 15 cm below a 250 W high-pressure mercury lamp with light wavelength from 350 nm to 450 nm which used as a light source. 4 mL MO solution was withdraw and measured its absorbance spectrum every 20 minutes using a UV-vis spectrophotometer (at λ = 464 nm) and poured back to the beaker after texting. It is noted that after every irradiation, the MO aqueous solution should be added deionized water to ensure it maintain the initial quality. Meanwhile, repeated tests were performed to examine the reusability of photocatalysts.

## Electronic supplementary material

Facile Synthesis ZnS/ZnO/Ni(OH)2 Composites Grown on Ni Foam: A Bifunctional Materials for Photocatalysts and Supercapacitors
