# Supplementary material for: Facile Synthesis ZnS/ZnO/Ni(OH)2 Composites Grown on Ni Foam: A Bifunctional Materials for Photocatalysts and Supercapacitors
Source: Sci Rep. 2017 Jun 8;7:3021. doi: 10.1038/s41598-017-03200-2 (PMC5465224; doi:10.1038/s41598-017-03200-2)
Supplement: Supplementary file 1 — Facile Synthesis ZnS/ZnO/Ni(OH)2 Composites Grown on Ni Foam: A Bifunctional Materials for Photocatalysts and Supercapacitors [file 41598_2017_3200_MOESM1_ESM.doc]

**Supporting Information**

Facile Synthesis ZnS/ZnO/Ni(OH)_2_ Composites Grown on Ni Foam: A Bifunctional Materials for Photocatalysts and Supercapacitors

Jin Hao^1^, Xiaobing Wang^1^, Fanggang Liu, Shuang Han*, Jianshe Lian*, Qing Jiang

Key Laboratory of Automobile Materials, Ministry of Education, and Department of Materials Science and Engineering, Jilin University, Changchun 130022, P.R. China

Table S1. The content of ZnS, ZnO and Ni(OH)_2_ in the NZZN-2mmol, NZZN-3mmol, NZZN-4mmol and NZZN-5mmol.

|  | **NZZN-2mmol** | **NZZN-3mmol** | **NZZN-4mmol** | **NZZN-5mmol** |
| --- | --- | --- | --- | --- |
| ZnS | 20.89 | 18.03 | 16.85 | 15.72 |
| ZnO | 68.18 | 76.24 | 78.37 | 79.62 |
| Ni(OH)_2_ | 10.93 | 5.73 | 4.78 | 4.66 |

Table S1 lists the content of ZnS, ZnO and Ni(OH)_2_ in the NZZN-2mmol, NZZN-3mmol, NZZN-4mmol and NZZN-5mmol attributed to the energy spectrum results. It can be found that the material content prepared by one step hydrothermal reaction is presented ZnO>ZnS>Ni(OH)_2_.


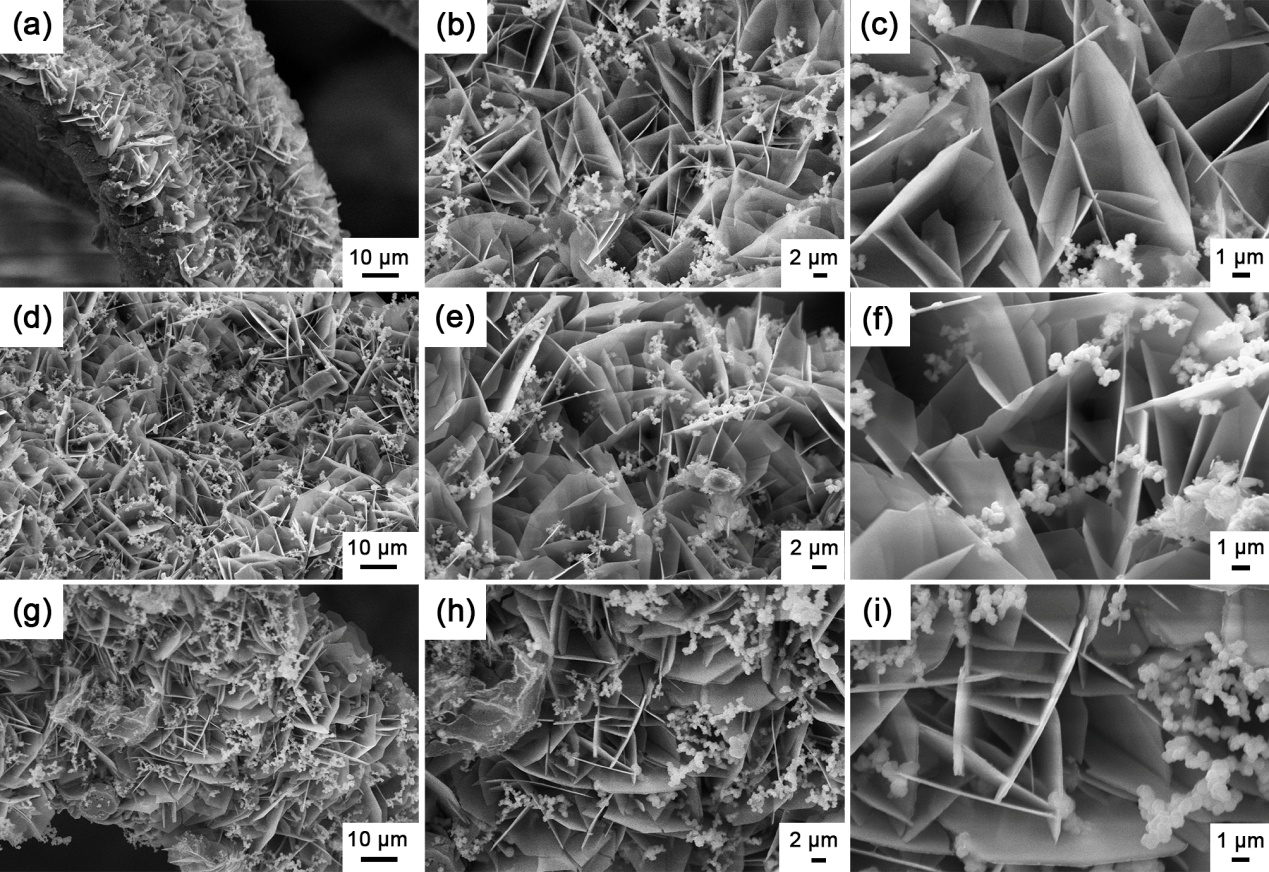


**Figure S1. FESEM images of (a, b and c)** **NZZN-2mmol, (d, e and f) NZZN-3mmol and (g, h and i) NZZN-5mmol.**

To illustrate the effect of Zn content on the morphology of NZZN, the samples contained NZZN-2mmol, NZZN-3mmol and NZZN-5mmol which prepared in different mole range of 2mmol, 3mmol and 5mmol of Zn(NO_3_)_2_ were characterized in FESEM images, as shown in Fig. S1. All pictures demonstrate a porous nanosheet networks structure with abundant of submicron particles were grown in Ni foam successfully. It is observed that the number of nanosheets and nanoparticals both increase and become more dense with the mole increase under the same magnification.


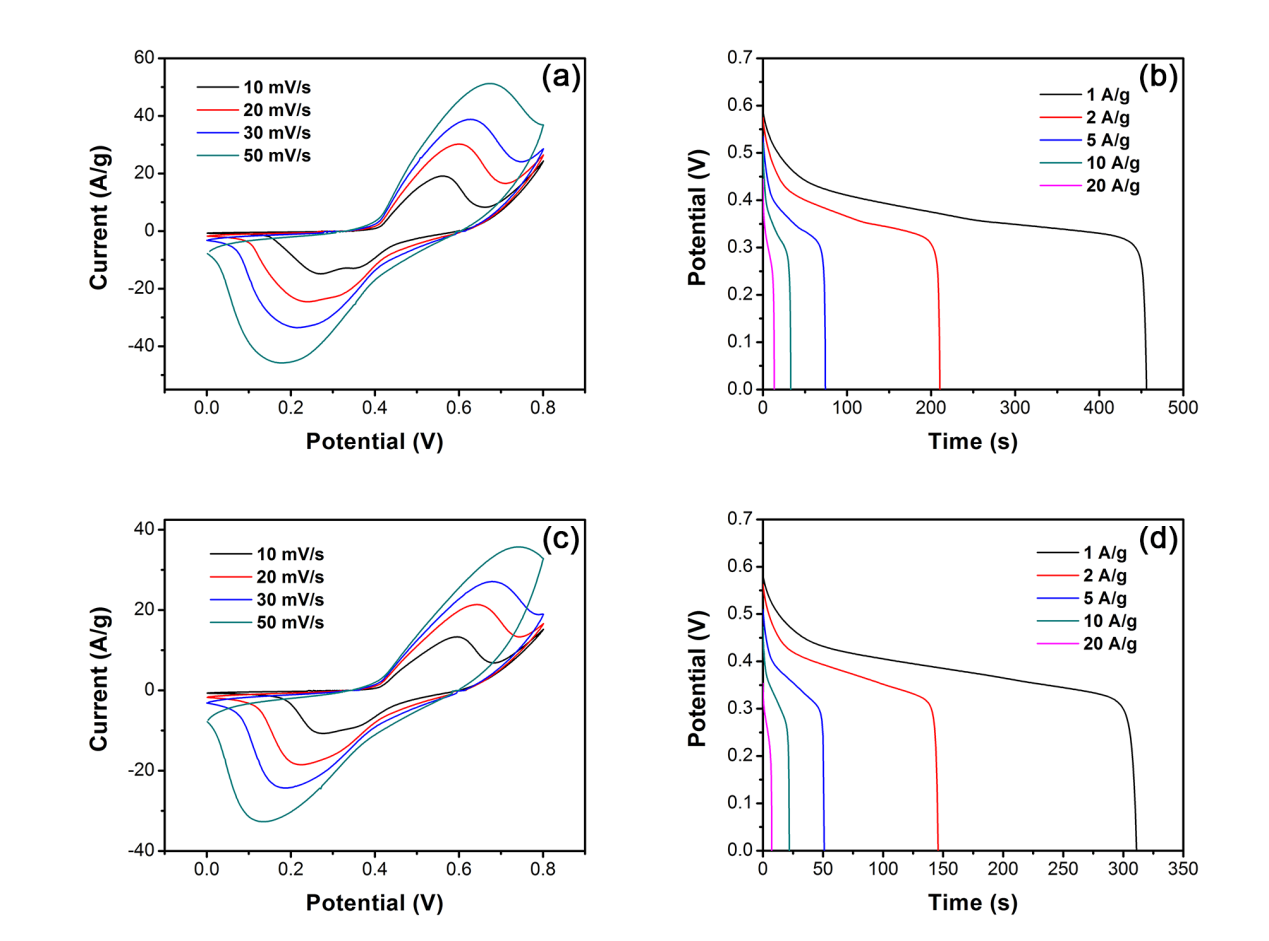
**Figure S2. Rate performance curves of (a and b) NZZN-3mmol and (c and d) NZZN-5mmol electrodes.**

Elctrochemistry tests including cyclic voltammetry (CV) and galvanostat charge-discharge measurements were carried out to further compare the rate performance of NZZN-3mmol and NZZN-5mmol electrodes, and the results are shown in Fig. S2 CV curves at different scan rates range of 10, 20, 30 and 50 mV s^-1^ of NZZN-3mmol and NZZN-5mmol electrodes are shown in Fig. S2a and Fig. S2c, we can find that NZZN-3mmol possesses larger area than NZZN-3mmol at the same scan rate. Fig. S2b and Fig. S2d show alvanostat charge-discharge curves of NZZN-3mmol and NZZN-5mmol electrodes from 0 to 0.61 eV over a range of current densities (1, 2, 5, 10 and 20 A g^-1^). Obvious discharge platforms are observed which ascribe to the pseudocapacitance behavior.

**Table S2.** Calculated values of R_s_, C_dl_, R_ct_, W and C_L_ of the supercapacitor electrodes consisting of NZZN-2mmol, NZZN-3mmol and NZZN-5mmol through fitting of the experimental impedance spectra based on the proposed circuit in Fig. 6e.

| **Samples** | **R_s_/ohm** | **C_dl_/F** | **R_ct_/****ohm** | **W/ohm** | **C_L_/F** |
| --- | --- | --- | --- | --- | --- |
| NZZN-3mmol | 0.3580 | 0.0231 | 0.0909 | 2.389 | 2.953 |
| NZZN-4mmol | 0.3163 | 0.0239 | 0.0887 | 2.055 | 2.706 |
| NZZN-5mmol | 0.4338 | 0.0237 | 0.0938 | 2.657 | 2.507 |

Nyquist plots were analyzed by the software of ZSimpWin on the basis of the equivalent circuit, and the results are shown in Table S2. It is observed that the NZZN-4mmol samples possesses the lowest R_s_ and R_ct_ values.

**
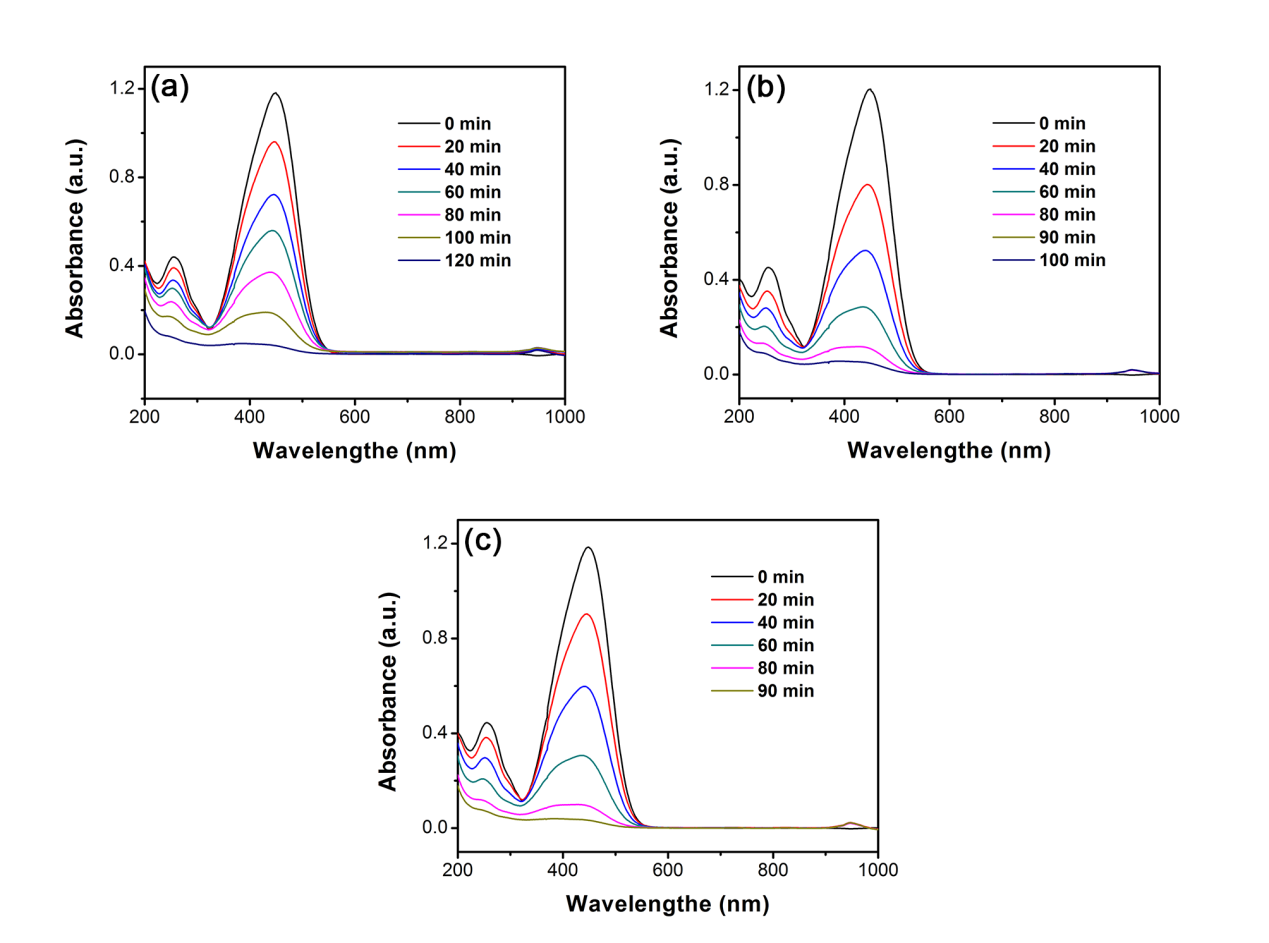
**

**Figure S3. UV-vis absorption spectra of MO solution with (a)** **NZZN-2mmol, (b) NZZN-3mmol and (c) NZZN-5mmol samples.**

Fig. S3 depicts the time progress of UV-vis spectra upon MO solution adsorption and photodegradation with NZZN-2mmol, NZZN-3mmol and NZZN-5mmol samples, croesponding to Fig. S3a-c, respectively. As we can see, under the visible light irradiation, the maximum absorbance of NZZN-2mmol almost disappeared in 120 minutes, which spend the longest time, while the sample NZZN-5mmol spend the least time for 90 minutes, suggesting that it presents the best photocatalytic performance among all the samples.


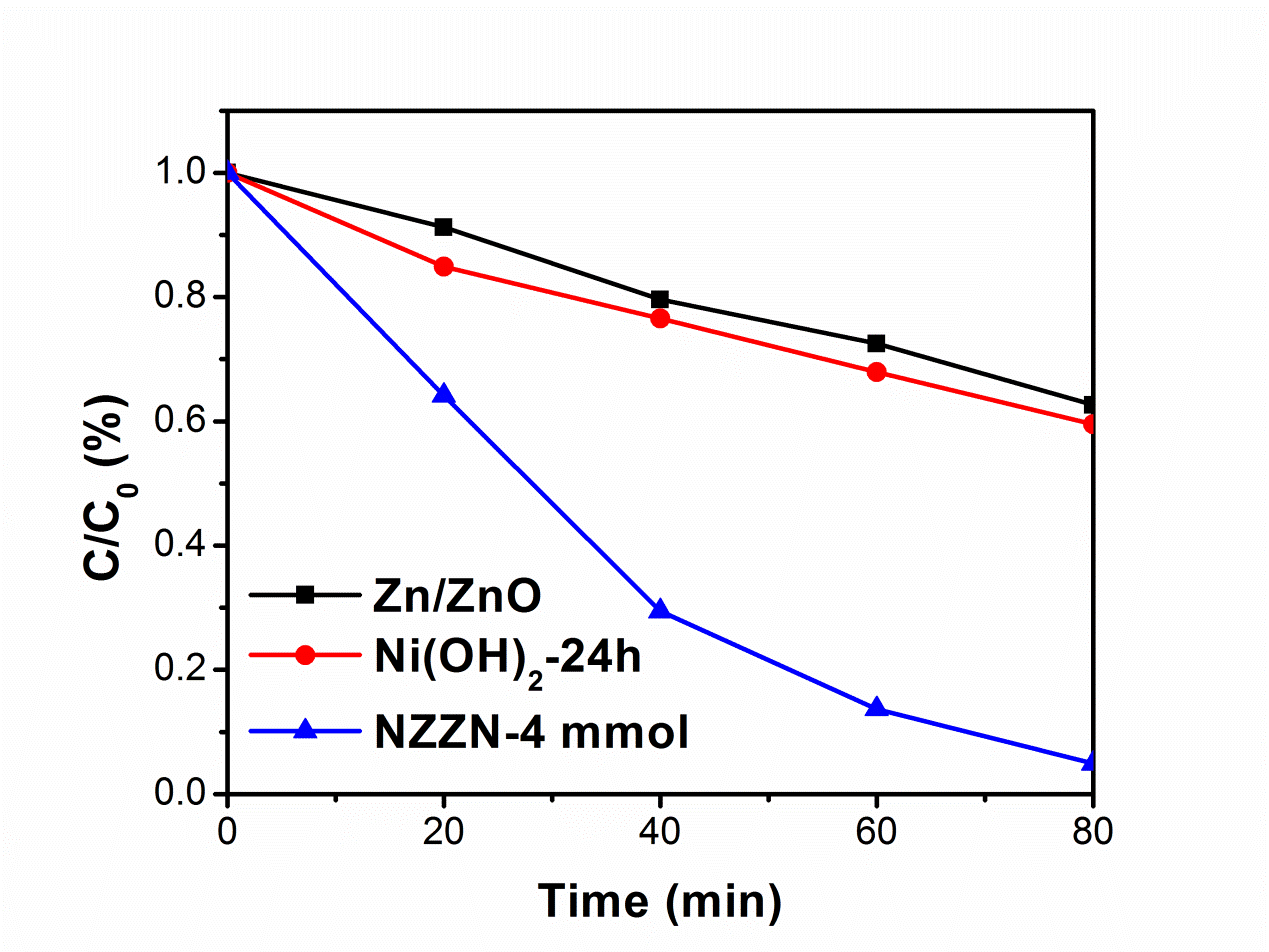


**Figure S4.** UV-vis absorption spectra of MO solution with as-obtained ZnS/ZnO, Ni(OH)_2_-24h and NZZN-4mmol.

As shown in Fig. S4, the photocatalytic efficiency of NZZN-4mmol is much higher than that of pure Ni(OH)_2_ and ZnS/ZnO.


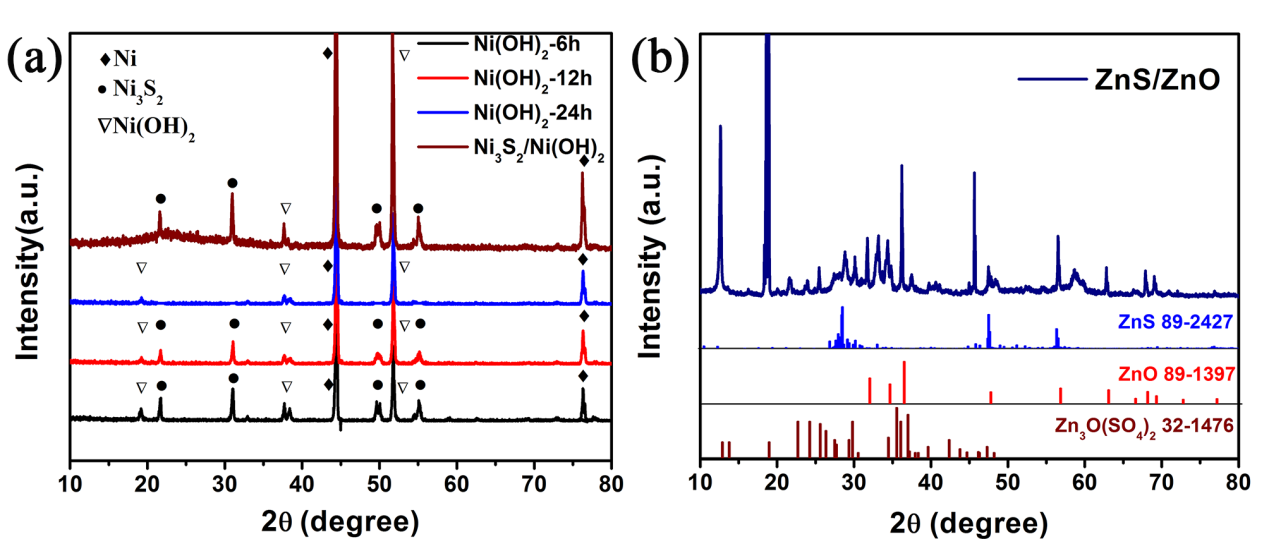


**Figure S5.** XRD patterns of (a) Ni_3_S_2_/Ni(OH)_2_, Ni(OH)_2_ and (b) ZnS/ZnO samples.

The XRD pattern, Figure S5a, shows the phase structure of products in different stages of hydrothermal reaction when synthesised the Ni(OH)_2_/Ni foam composites. There are diffraction peaks indexed to Ni (JCPDS card no.70-1849), Ni_3_S_2_ (JCPDS card no.85-1802) and Ni(OH)_2_ (JCPDS card no.74-2075) for Ni_3_S_2_/Ni(OH)_2_, Ni(OH)_2_-6h and Ni(OH)_2_-12h samples except Ni(OH)_2_-24h which lack of Ni_3_S_2_, demonstrating that the pure Ni(OH)_2_ was prepared via secondary hydrothermal method. As shown in Figure S5b, The diffraction peaks of Zinc compound could be assigned to the ZnS (JCPDS card no.89-2427) and the ZnO (JCPDS card no.89-1397). The diffraction peaks show the inevitably presence of sulfide impurities during hydrothermal processes.
